# How vascular smooth muscle cell phenotype switching contributes to vascular disease

**DOI:** 10.1186/s12964-022-00993-2

**Published:** 2022-11-21

**Authors:** Genmao Cao, Xuezhen Xuan, Jie Hu, Ruijing Zhang, Haijiang Jin, Honglin Dong

**Affiliations:** 1 Department of Vascular Surgery, The Second Hospital of Shanxi Medical University, No. 382, Wuyi Road, Taiyuan, China; 2 Department of Nephrology, The Second Hospital of Shanxi Medical University, No. 382, Wuyi Road, Taiyuan, China

**Keywords:** Vascular smooth muscle cells, Phenotype switching, Atherosclerosis, Vascular aging, Aortic aneurysm

## Abstract

Vascular smooth muscle cells (VSMCs) are the most abundant cell in vessels. Earlier experiments have found that VSMCs possess high plasticity. Vascular injury stimulates VSMCs to switch into a dedifferentiated type, also known as synthetic VSMCs, with a high migration and proliferation capacity for repairing vascular injury. In recent years, largely owing to rapid technological advances in single-cell sequencing and cell-lineage tracing techniques, multiple VSMCs phenotypes have been uncovered in vascular aging, atherosclerosis (AS), aortic aneurysm (AA), etc. These VSMCs all down-regulate contractile proteins such as α-SMA and calponin1, and obtain specific markers and similar cellular functions of osteoblast, fibroblast, macrophage, and mesenchymal cells. This highly plastic phenotype transformation is regulated by a complex network consisting of circulating plasma substances, transcription factors, growth factors, inflammatory factors, non-coding RNAs, integrin family, and Notch pathway. This review focuses on phenotypic characteristics, molecular profile and the functional role of VSMCs phenotype landscape; the molecular mechanism regulating VSMCs phenotype switching; and the contribution of VSMCs phenotype switching to vascular aging, AS, and AA.

**Video Abstract**

**Supplementary Information:**

The online version contains supplementary material available at 10.1186/s12964-022-00993-2.

## Introduction

Over the last three decades, cardiovascular disease (CVD) has been reported as the primary cause of morbidity and mortality worldwide [1, 2]. The pathophysiological basis of vascular diseases involves a wide range of mechanisms including vascular aging, calcification, atherosclerosis, extracellular matrix (ECM) remodeling, and immune cells infiltration. VSMCs are the most abundant cell type in vessels that not only exert vascular physiological function but also contribute to vascular diseases in varied ways. Recent studies indicated that VSMCs display a high degree of plasticity and possess multiple differentiation potentials in disease states, finally achieving unexpected phenotypes with changes in morphology and function. It is interesting to note that VSMCs phenotype transformation could be multi-directional in a single local lesion. According to literature reports, the process involves metabolism dysfunction, stress response, inter-cellular communication, and multi-level signaling pathways, which together constitute a sophisticated regulatory network. Due to the significant correlation between VSMCs phenotype and the occurrence and progression of vascular diseases, manifold studies have pointed out that VSMCs phenotype switching underlies the progression of vascular diseases, and found that inhibition of VSMCs switching from contractile to other phenotypes helps alleviate the severity of vascular diseases. Studies on VSMCs phenotype switching not only elucidate a new mechanism of vascular diseases but also provide a new therapeutic target. This review summarizes the characteristics and functions of existing VSMCs phenotypes, elaborates factors driving VSMCs phenotype switching, and elucidates the contribution of various VSMCs phenotypes to vascular diseases. We also prospect the potential of VSMCs phenotype switching as a treatment for vascular diseases.

## Normal physiological function of VSMCs

Anatomically, a healthy large artery is composed of a three-layer loop structure: (1) tunica intima consisting of a single layer of endothelial cells; (2) tunica media consisting of multiple VSMCs layers, and elastic fiber layers between VSMCs layers; (3) tunica adventitia containing adipocytes, fibrous connective tissue, and ECM [3]. The elasticity of artery mainly credits to active contraction of VSMCs and passive contraction of elastic lamellae that composed of collagen and elastin fibers. The contractile properties allow VSMCs to regulate vessel diameter, blood pressure, and blood flow distribution. Regarding VSMCs located in aorta, they are exposed to high pressure, shear stress, and pulsatile blood flow [4]. This additionally requires a considerable stiffness level of VSMCs.

In large arteries, the VSMCs contract to maintain the normal shape of the artery during systolic ejection period. On the other hand, VSMCs in small resistance arteries are responsible for regulating blood flow distribution, reducing the diameter of the small resistance artery [5]. VSMCs contractility was regulated by active substances secreted by endothelial cells, autonomic nervous system, adrenal hormone, vasoactive peptides, and ROS. The contraction of VSMCs is triggered by ionized calcium influx. Calcium-calmodulin-mediated activation of myosin light chain phosphorylation and actin-myosin cross-bridge cycling act in concert to facilitate myofilament contractility [6]. Additionally, actin cytoskeleton modulates VSMCs contractility by communicating with ECM. Actin filaments are bound to talin and vinculin, indirectly linked to integrin β subunit [7]. Thus, contractile force in actin filaments could be conveyed to integrin receptors and then ECM through inside-out signaling. Similarly, extracellular segments of integrins bind to ligands in the ECM and transmit extracellular signals or force to actin cytoskeleton through out-inside signaling. Detailed mechanisms have been described in an excellent review [4].

## The landscape of VSMCs phenotypes

### The high plasticity of VSMCs

In healthy adults, the vast majority of VSMCs are located in tunica media and represent quiescent contractile phenotype. Whereas, contractile VSMCs spontaneously modify their phenotype instantaneously to a highly synthetic phenotype when the vessel is physically or chemically damaged. VSMCs phenotype switching serves as a vital step in the repair process of vascular damage. As synthetic VSMCs undergo down-regulation of contractile gene expression, cytoskeleton remodeling, and cell reprogramming, VSMCs dramatically increase their proliferation capacity and secret matrix metalloproteinases to promote extracellular matrix remodeling. The acquired proliferation and migration capacity of synthetic VSMCs benefits neointima formation following vascular injury. After injury is released, locally dysfunctional signaling pathways within the vessels restores homeostasis and the VSMCs regains their contractile phenotype/characteristics.

The potential plasticity shown by VSMCs is comprehensive and inherent, since VSMCs necessitate high abilities of proliferation, migration, and ECM secretion (such as collagen and elastin) in response to vascular morphogenesis and frequent vascular injury. However, the high plasticity of VSMCs may make it susceptible to harmful environmental stimuli, thus transforming into pathogenic phenotypes. VSMCs phenotype switching is usually triggered and regulated by growth factor/inhibitors, transcription factors, mechanical force, cell–cell, and cell–matrix interactions, as well as a variety of inflammatory mediators [8].

### Synthetic VSMCs

The synthetic VSMCs are considered as the de-differentiated state of contractile VSMCs, accompanied by morphologic changes from spindle shape to irregular shape. With the decrease of contractile protein expression, the proliferation and migration ability of synthetic VSMCs was enhanced, which played a role in repairing vascular injury. And true to its name, synthetic VSMCs secrete large amounts of collagen, elastin, and matrix metalloproteinase (MMP) causing vascular ECM remodeling. Therefore, synthetic VSMCs almost exist in all types of vascular diseases such as atherosclerosis, aneurysm, neointima. Synthetic VSMCs are prone to further differentiate into alternative phenotypes such as macrophage-like and osteogenic VSMCs. Although some scholars believe that there is no convincing marker for the synthetic VSMC, numerous studies have confirmed that osteopontin (OPN), MMP-9, epiregulin (EREG), and vimentin (VIM) are highly expressed in the synthetic VSMC [9, 10]. A wide range of regulatory factors and signaling pathways are involved in the transition from contractile to synthetic VSMC, including metabolic factors, growth factors, non-coding RNA, immune-inflammatory molecules and so on. They will be discussed in detail in the next section.

### Osteogenic VSMCs

Vascular calcification (VC) is a common sign in the aged population and chronic kidney disease (CKD) population, which could consequent in increased artery hardness, impaired elasticity, and deficient compliance in advanced stage. VC can be regarded as a process of osteogenesis since the activation of osteogenic genes in the vascular cells play a pivotal role. Recent studies have gradually pointed out the fact that VSMCs switching to the osteogenic phenotype is the principal reason for vascular calcification [11–13]. Osteogenic VSMCs are also called osteoblast-like VSMCs because of their similarity to osteoblast (see Table 1). During the transformation to the osteogenic phenotype, contractile markers are down-regulated and bone markers such as OC, collagen type I alpha 1 (Col1α1), collagen type II, X (Col II, X), OPN, BMP2, and alkaline phosphatase (ALP) are progressively expressed [12, 14, 15]. We have learned that metabolic factors promoting osteogenic VSMCs incorporate high phosphorus level, high calcium level, oxidized lipoproteins, and reactive oxygen species (ROS). High-phosphate environment is the primary cause of vascular calcification, as it is commonly observed in chronic kidney diseases patients with disturbances in mineral and bone metabolisms [16].

**Table 1:** Osteoblast versus osteogenic VSMCs

	Osteoblasts	Osteogenic VSMCs	Reference (PMID)
Origin	Chondrocytes Marrow stromal cells Bone-lining cells Fibroblasts	Dedifferentiated VSMCs	34213032
Synthesis secretion and mineralization of bone matrix	Yes	Yes	34213032
Markers	ALP, BSP, OCN, OPN, COL I	OCN, OPN, ALP, COL I, COL II, COL X,	29514202
Transcription factors	RUNX1, RUNX2, SOX11, MSX1, MSX2, Dlx5, DLX6, AP1, KNOX-20, Sp3, Sp7(OSX), ATF4, KLF2, KLF4	RUNX2, SOX9, KLF4, MSX1, MSX2	32571873 16795049 21703370 22952236 23888050 21898406 33608506 16795049
Location	Bone tissue	Media Intima Necrotic core	32674599

ALP, alkaline phosphatase; BSP, bone sialoprotein; OCN, osteocalcin; OPN, osteopontin; COL, collagen; RUNX, runt-related transcription factor; OSX, osteoblast-specific transcription factor osterix; SOX, SRY-Box transcription factor; MSX, msh homeobox; DLX, distal-less homeobox; AP, activator protein; SP, SP transcription factor; ATF, activating transcription factor; KLF, kruppel-like factor

### Macrophage-like VSMCs

Macrophage-like VSMCs are termed for their similar surface markers and function with macrophages (see Table 2). Macrophage-like VSMCs demonstrate low expression of contractile markers and possess functions similar to macrophages such as innate immune signaling, phagocytosis, and efferocytosis [17]. As early as 1997, Andreeva et al. identified cells co-expressing CD68 and α-SMA in human aorta atherosclerosis plaque, especially in lipid-rich regions [18]. Subsequently, Allahverdian et al. estimated that these chimeric cells accounted for about 40 percent of all CD68-positive cells in atherosclerotic lesions. However, these studies could not determine whether chimeric cells were VSMC-derived cells that activated macrophage markers, or macrophages that activated SMC markers, or neither, until cell lineage tracing strategy confirmed that these chimeric cells are VSMCs-derived [19].

**Table 2:** Macrophages versus macrophage-like VSMCs

	Macrophages	Macrophage-like VSMCs	Reference (PMID)
Origin	Myeloid monocytes	Dedifferentiated VSMCs	
Phagocytosis	Yes	Yes	26892967
Cholesterol efflux	Yes	Yes	24481950
Antigen presenting	Yes	Unclear	30928403
Cytokines secretion	**M1:** IL-1, IL-6, TNF-α, CXCL9, CXCL10, CXCL11 **M2:** IL-1RA, IL-10, CCL2, CCL7, CCL17, CCL18, CCL23	TNF-α, IL-1b, IL-6, IL-8, IL-17, CCL2, CCL7	25070003 24481950
Markers	M1: CD14, CD68, CD80, CD86 M2: CD14, CD68, CD206, CD163	CD68, MAC-2 (LGALS3) ACTA2, MYH11	25070003
Transcription factors	STAT family PPARγ C/EBP family IRF family	KLF4	22025054
Location	Tunica intima Tunica media Tunica adventita Fibrous cap Necrotic core	Tunica media Tunica intima Fibrous cap Necrotic core	25070003 24481950 32895539 24685316

STAT, signal transducers and activators of transcription; PPARγ, peroxisome proliferator-activated receptor gamma; C/EBP, CCAAT/enhancer-binding protein; IRF, interferon regulatory factors; TNF-α, tumor necrosis factor α; IL, interleukin; CXCL, C-X-C motif chemokine ligand; CCL, C–C motif chemokine ligand; M1: classically activated macrophages; M2: alternatively activated macrophages

The phagocytosis of VSMCs in atherosclerosis relies on its macrophage-like phenotype, so high oxLDL and Cholesterol are the primary metabolic factors driving macrophage-like VSMCs [18, 20]. Furthermore, age-enhanced oxLDL can also promote VSMCs to obtain the phenotype and function of macrophages [21]. However, the proportion of macrophage-like VSMCs in the pathogenesis of AS is lower than that of macrophage itself, and the level of the inflammatory factors in macrophage-like VSMCs is also inferior to that of monocyte-derived macrophages [17]. ATP-binding cassette transporter 1 (ABCA1) and ATP binding cassette transporter G1 (ABCG1), genes that regulate cholesterol efflux, are activated when VSMCs engulf oxLDL and cholesterol [17]. Enhancing expression of ABCA1 and ABCG1 may reduce the burden of macrophage and VSMCs [17]. However, the available evidence is insufficient to propose that macrophage-like VSMCs adopt the full function of a macrophage. Therefore, further studies need to investigate whether macrophage-like VSMCs represent antigen, mediate innate immune response, or exert anti-inflammatory effects (like M2 macrophage).

### Mesenchymal-like VSMCs

In fact, there is no uniform official definition of mesenchymal-like VSMCs. The VSMCs that express partial mesenchymal markers are referred to mesenchymal-like VSMCs. Yap et al. defined mesenchymal-like VSMCs as VSMCs expressing stem cell marker SCA1 with similar functions to mesenchymal stem cells [22]. This includes pioneer cell phenotype identified by Alencar et al. [23] and SEM cells (SEM = stem cell + endothelial cell + monocyte) identified by Pan et al. [24]. In 2004, Hu et al. initially discovered SCA1+ CD34+ cells in the tunica adventitia of aortic root of ApoE−/−mice [25]. These SCA1+ CD34+ cells were termed as AdvSca1 progenitors as they could differentiate into contractile VSMCs. Majesky et al. used Myh11-CreERT2-LacZ mice and SM22α-CreERT2-LacZ mice for cell-lineage tracing and found that approximately 8% of SCA1+ CD34+ adventitial progenitors were derived from contractile VSMCs in tunica media, and could subsequently differentiate into macrophage-like (F4/80+), adipocyte-like (FABP4+), and endothelial-like (VWF+) VSMCs [26].

Single-cell sequencing techniques and lineage-tracing experiments revealed the presence of mesenchymal-like VSMCs in mice atherosclerotic plaques [27], normal AA [28], and AA in Marfan's syndrome (MFS) [29]. Mesenchymal-like VSMCs is functionally similar to synthetic VSMCs: both of them have strong proliferative capacity and the ability to switch into other phenotypes of VSMCs for repairing vascular injury. Artery injury and KLF4 activation induced the expression of mesenchymal markers (such as stem cell anti-1 (SCA1)/LY6A, CD34, and CD44) in VSMCs, and down-regulated contractile protein expression [22]. In addition to deriving from contractile VSMCs, Mesenchymal-like VSMCs can also derive from SCA1+ vascular stem cells (VSCs) originally located in the tunica adventitia. Once vascular injury occurred, VSCs migrate to the tunica media and differentiate into mesenchymal-like VSMCs for repairing [30].

The role of mesenchymal-like VSMCs in arterial disease is uncertain. Earlier studies believed that tunica adventitia derived mesenchymal-like VSMCs contribute to atherosclerotic plaque growth [25] and CKD-induced vascular calcification [31]. Whereas, a recent study found that adventitial VSCs did not differentiate into the pathogenic VSMCs in atherosclerosis [32]. Pan et al. indicated that mesenchymal-like VSMCs can be induced into macrophage-like VSMCs, or into contractile VSMCs, depending on external stimulus conditions [24]. Finally, the role of mesenchymal-like VSMCs in AA has been rarely studied.

### Fibroblast-like VSMCs

Single-cell transcriptome revealed that Fibroblast-like VSMCs are present in atherosclerosis plaque [23], AA in MFS [33], and normal AA [34]. Nevertheless, the markers used to mark fibroblast-like VSMCs and mesenchymal-like VSMCs in single cell sequencing overlapped, creating confusion over their definition. Unique markers of fibroblast-like VSMCs include fibroblast markers lumican (LUM), biglycan (BGN), and decorin (DCN), and share the same marker SCA1/LY6A with mesenchymal-like VSMCs [35]. In respect of the gene expression profile displayed by single-cell transcriptome, fibroblast-like VSMCs perform three main functions: synthesizing ECM, enforcing cell–matrix adhesion, and promoting cell proliferation. Fibroblast-like VSMCs switching is associated with arterial fibrosis resulting in increased arterial stiffness [36]. Interestingly, given that switching of VSMCs from contractile to other phenotypes requires turning on the KLF4 switch, nevertheless, the adventitial SCA1+ progenitor (AdvSca1) cells need to inactivate KLF4 for obtaining fibroblast-like phenotype and contributing vascular fibrosis [37]. This may be the result of a higher differentiation degree of fibroblast-like VSMCs than AdvSca1 cells if we consider KLF4 as an indicator of the degree of differentiation of VSMCs. Cholesterol-induced VSMCs phenotype switching also incorporate fibroblast-like VSMCs in addition to macrophage-like VSMCs [37]. This is because high extracellular Cholesterol would lead to Cholesterol overload of endoplasmic reticulum (ER) in VSMC, resulting in unfolded protein response (UPR), phosphorylation of Ire1α and eIF2α, and increasing of Atf4, Atf6, Grp78, Grp94, and Edem protein levels. The appearance of fibroblast-like VSMCs explained the formation of fibrous cap in AS plaque. It is not clear whether fibroblast-like VSMCs are derived from mesenchymal-like VSMC, or mesenchymal-like is a prophase transition state of fibroblast-like VSMC, but their common marker, SCA1/LY6A, raises the possibility.

## Factors that contribute to VSMC phenotypes switching

### Metabolic factors

Dysregulation of metabolic homeostasis could be the initial reason for VSMCs phenotype switching. When VSMCs were cultured in vitro with high lactate concentration, their proliferation and migration ability increased, accompanied by inhibited contractile markers, enhanced synthetic markers, and irregular morphological changes [38]. This may be the consequence of lactic acid enhancing monocarboxylic acid transporters (MCTs) and NDRG3 expression [38]. High calcium and high homocysteine could promote arteriosclerosis by up-regulating BMP2, OC, and down-regulating of OPN. This may be the reason why AS plaques are prone to calcification [39]. Additionally, high homoarginine reinforces VSMCs osteogenic transition and vascular calcification given the background of hyperphosphatemia [40].

Oxidized low density lipoprotein (ox-LDL) is a canonical atherosclerosis risk factor. Manifold studies have supported that ox-LDL induces vascular calcification by augmenting VSMCs osteogenic transformation, in which TLR4/NF-Κb, TGF-β, RUNX2, and ERK1/2 signaling are involved [41–45]. Furthermore, the capacity of promoting vascular calcification could be magnified by vascular peroxidase 1 (VPO1) [46]. Enzyme modified non-oxidative LDL (ELDL) is another circulating factor contributing to the transformation of VSMCs to osteogenic phenotype by up-regulating expression of DMP-1, SPP1, SP7, and BMP2 [47]. ELDL is more likely to induce VSMCs conversion to foam cells than oxLDL and natural LDL because of its greater capacity of upregulating ANGPTL4 [47]. In Lucinda et al., high circulating estrogen level promotes osteoblast marker expression in VSMCs by inhibiting estrogen receptor α/β [48], which is consistent with the clinical study that estrogen is associated with higher vascular calcification incidence [49]. High Cholesterol up-regulates macrophage markers CD68 and Lgals3 and promotes up-regulation of SREBP family, PPARγ, NFKB, and KLFs pathways [17, 20, 37].

As diabetes mellitus (DM) is a major risk factor for atherosclerosis [50], advanced glycation end products (AGEs) increase arterial wall stiffness by cross-linking with elastin and collagen, or accelerate foam cell formation by cross-linking with oxLDL [51]. AGEs promote the transformation of macrophages into pro-inflammatory M1 subtype in plaques and reduce VSMCs contractile protein expression through the Notch pathway [52].

### Growth factors/cytokines

Growth factors are a group of canonical molecules that regulate VSMCs phenotype. Platelet-derived growth factor BB (PDGF-BB) is the first growth factor found to regulate the dedifferentiation of VSMCs [53]. PDGF-BB is often used in experiments to study the characteristics of synthetic VSMC [54, 55]. Transforming growth factor-beta (TGF-β) is widespread in biological responses and plays a pivotal role in the development and maturation of vascular system. TGF-β reversed synthetic VSMCs into functional contractile VSMCs through increasing the interaction between downstream phosphorylated SMAD 2/3 and SMAD consensus binding sites of contractile markers gene such as SM actin, calponin1, smooth muscle protein 22-alpha (SM22α) [56]. In addition, fibroblast growth factor (FGF) acts as an antagonist of TGF-β to inhibit the expression of contractile markers [57]. Evidence has indicated that TGF-β signal intensity was directly proportional to the degree of differentiation of VSMCs, while FGF signal intensity was inversely proportional to it. TGF-β could also enhance microRNA-143/145 to inhibit klF4-induced synthetic transformation [58]. Nesfatin-1, an adipocytokine function as elevated blood pressure [59], was also found to trigger VSMCs switching to synthetic state and promote VSMCs proliferation capacity [60].

### Transcription factors

Recent data has illustrated that multiple transcription factors manipulate transcript level of myogenic determination protein. Among these transcription factors, KLF4 is the most reputed one regulating VSMCs synthetic transformation and is involved in the transformation to most VSMCs phenotypes [22, 27]. ChIP-sequence of mice atherosclerosis lesions indicated that KLF4 targets genes regulating epithelial adherens junction, integrin signaling, leukocyte recruitment, actin regulation, and ECM organization [61]. KLF4 inhibits the expression of VSMCs contractile marker genes through various distinct mechanisms. There are two KLF4 binding sites in the SM22α promoter region: (^−263^CACCC^−259^) and (^−136^GTGGG^−132^). CC(A/T-rich)_6_GG (CArG) are widely present in promoter enhancer region of contractile gene [62]. Serum response factor (SRF) directly binds to them for activating muscle-specific transcription [63]. KLF4 inhibits SRF and myocardin binding to CArG box chromatin KLF4 [64]. In addition, KLF4 also affects contraction protein expression through epigenetic modifications such as posttranslational histone modifications. Oliver et al. Showed that cardiomyin enhanced the connection between SRF and CArG box by enhancing histone methylation [65]. Conversely, KLF4 enhanced deacetylation of histone H4 at the contraction gene and prevented SRF from binding to CArG Box [65].

Runt-related transcription factor 2 (RUNX2), an essential osteogenic transcription factor for bone formation, is also an early expressed regulator driving the osteogenic transition of VSMCs [66]. RUNX2 regulates numerous target genes, including bone matrix proteins COL1A1, SPP1, IBSP, BGLAP2, and FN1, and also regulates the signal pathways related to osteoblast differentiation and osteoblast progenitors proliferation [67]. RUNX2 is high expressed in calcified arteries of mammals and human sapiens, and knocking-out RUNX2 significantly reduces vascular calcification and formation of osteogenic VSMC [13, 68]. Elevated circulating phosphate (uptake by VSMCs via Pit1 and Pit2), sodium-dependent phosphate transporters, oxidative stress, and aldosterone drive RUNX2 expression [69–71]. DNA-damage increases RUNX2 expression, RUNX2 PARylation, and RUNX2 binding to osteogenic gene promoter regions, leading to vascular calcification in aging vessels. The RUNX2 PARylation, in turn, promotes the apoptosis of DNA damaged VSMCs, further promoting vascular aging [72]. BMP2, ERK/MAPK, and PI3K/AKT signaling pathways affect the post-translational modification of RUNX2 (including phosphorylation, acetylation, ubiquitination, and O-GlcNAcylation), which indirectly regulates the transcription level of osteogenic genes [66]. TNF ligand-related molecule 1A (TL1A) exerts protective properties in atherosclerosis through inhibiting VSMCs switch to osteogenic phenotype and synthetic phenotype [10]. This may be attributed to TL1A lowering RUNX2 and ALP expression and causing RUNX2 nuclear translocation. Given that miR-203-3p inhibits RUNX2 to play a role in inhibiting osteogenic differentiation, TL1A increased miR-203-3p expression to prevent VC.

The transcription factor SOX9 serves as a “master switch” in chondrogenesis, it combined in cartilage cells to the promoter region of COL2A1 COL11A2, ACAN, and CDRAP for promoting cartilage formation [73]. SOX9 exerts a VC-inhibiting effect because SOX9 binds directly to RUNX2's runt domain and inhibits RUNX2 function by its evolutionarily conserved high-mobility group [74]. Cheng et al. Further demonstrated that SOX9 and RUNX2 had an opposite expression trend, and SOX9 enhanced RUNX2 degradation through the lysosomal pathway [75]. KLF4 is also very important for osteogenic differentiation. High phosphate could increase expression of KLF4 and KLF4 is elevated in calcified aorta. KLF4 not only binds to CArGs/TCE-containing promoter regions of α-SMA and SM22α to suppress their transcription, but also promotes OPN transcription level [61].

### Non-coding RNAs (ncRNAs)

Emerging studies have shed light on the importance of miRNAs in regulating VSMCs phenotype switching: miRNA can independently regulate the translation of markers mRNA, or become a key effector node in the phenotypic pathway regulated by other phenotypic factors. miR-182-3p directly binds to the 3-UTR of MYADM and inhibits its expression, and it was demonstrated that ADMA up-regulated the expression of VSMCs contraction marker by down-regulating miR-182-3p, and finally formed ADMA/miR-182-3p/MYADM pathway. miRNA let-7g directly binds to 3’-UTRs of PDGF-BB and MEKK1 mRNAs that inhibit ERK/KLF4 signaling pathways, thereby restraining VSMCs switching to synthetic phenotype [76]. miR-206 is another miRNA that powerfully maintains VSMCs contractile phenotype. Endothelial cell-derived miR-206 up-regulated the levels of α-SMA, SM22α, and CNN in VSMCs [77]. Furthermore, miR-206 forms a circuit with cardiomyin and Cx43 to regulate the phenotypic switch of VSMCs [78]. Overexpression of miR-206 in mouse pulmonary hypertension also increased levels of α-SMA and calponin [79]; On the other hand, miR-206 can directly inhibit KLF4 expression in cells except for VSMCs [80, 81], suggesting a protective role in vascular disease. In particular cases, miRNAs are encapsulated in exosomes secreted by non-VSMCs cells and reach the lesion site through the circulatory system, achieving the effect more specifically [82]. The extracellular vesicle of the endothelial cells (ECs) induces artery inflammation and promotes the hypertrophy and aging of VSMCs [83]. The process that secreting exosomes from ECs to VSMCs for phenotype switching could be prevented by miR-206 [77].

Early studies have established the importance of miRNAs in vascular calcification, and miRNAs can serve as vascular calcification markers [84]. In general, miRNAs that inhibit VSMCs ossification include miR-542-3p [85], miR-133a [89], miR-135a [86] miR-204 [87], and miR-223-3p [88], all of which regulate osteogenic switching of VSMCs by directly binding to osteogenic genes or related transcription factors. MiRNAs appear to play an intermediate role in the overall VSMCs phenotype regulatory network, rather than being the primary cause of VC. Given the diversity of miRNAs involved in VSMCs phenotype regulation, we believe that manifold miRNAs that potentially regulate VSMCs phenotype switching remain undiscovered.

### Immune and inflammatory molecules

Immune and inflammatory molecules are highly expressed in canonical vascular diseases and promote the infiltration of immune cells into the vascular wall. However, recent studies have found that immune and inflammatory molecules can promote the transformation of VSMCs into a pathogenic phenotype, which has become a new pathogenic mechanism. CD137 constitutes the TNF-receptor (TNFR) superfamily and interacts with CD137L to activate T-cell mediated immune response [89]. Recent research elaborated the mechanism how CD137-CD137 interaction mediated VSMCs phenotype switching makes artery prone to form neointima. The ability of CD137 to promote phenotype transformation of VSMCs is realized through its direct bind to NFATc1 protein since NFATc1 inhibits the expression of VSMCs contraction protein [89]. In the complement-mediated immune reaction, complement 3 (C3) reduced the VSMCs contractile markers expression, up-regulated the VSMCs synthetic markers expression, reduced the contractile force of VSMCs, and the ability of miR-145 to inhibit the formation of synthetic VSMCs has partially relied on inhibition of C3 [90]. Similarly, NLRP3 inflammasome and the included IL-1β convert VSMCs to synthetic phenotype [91]. Extracellular vesicles (EVs) secreted by pro-inflammatory macrophages accumulated in the vascular wall can also promote the dedifferentiation of VSMCs [92].

### Integrin family

Integrins mainly mediate signals from ECM to regulate the VSMCs phenotype. As heterodimer transmembrane receptors, integrins play a pivotal role in VSMCs biology by connecting intracellular actin cytoskeleton with ECM proteins. The integrin family consists of 18 α and 8 β subunits, and 24 distinct αβ heterodimers combinations have been identified [93]. Numerous assembled focal adhesion (FA) is one of the characteristics of contractile VSMCs. In MFS, synthetic VSMCs show an increase in the number of FA in individual cells but a decrease in FA density [29]. Integrin-based focal adhesion regulates the strength of cell–matrix connections, reshapes cells, senses chemical and physical stimuli in the extracellular environment, and acts as a signal transduction pathway for both inside-out and out-inside directions. For example, oligomeric matrix protein (COMP) degrades in the presence of vascular injury and promotes VSMCs dedifferentiation, whereas high COMP expression inhibits PDGF-BB-induced VSMCs dedifferentiation [94]. That is, COMP binds directly to Integrin α7β1 to induce focal adhesion proteins (including paxillin, vinculin, and talin) migrate from peri-nuclear region to cell membrane to assemble more focal adhesion and connect with actin skeleton and assemble into Parallel stress fibers. Integrin α8, when combined with β1, plays a similar role to Integrin α7β1 [95]. α9 subunit binds only to β1, and is involved in PDGF-BB-induced synthetic VSMCs transformation through interacting with Fibronectin-EDA to activate FAK/Src signaling and its downstream targets (including P-ERK, P38 MAPK, and P-GSK3 β/β-catenin signaling). Thus, mice with VSMC-specific knockdown of integrin α9 demonstrated the ability to resist neointima formation in vascular injury [96].

### Notch signaling pathway

Notch signaling is one of the most critical pathways that extensively determines the fate of multiple cell types. Notch Signaling regulates vascular morphogenesis during development, maintains vascular cell homeostasis, and mediates vascular repair after injury, which to some extent depends on the regulation of VSMC phenotype [97]. There are four receptors (Notch-1, -2, -3, and -4) in Notch pathway waiting for interaction with five ligands (Delta like 1, -3, -4, Jagged-1 and -2) [98]. Notch intracellular domain (NICD) of the receptor is shed into the nucleus and cooperates with CBF-1/ RBP-JK to up-regulate target genes. Known vascular-associated target genes are hairy and enhancer of split (HES) and HES-related repressor protein (HERP). Subsequently, HES and HERP exert Notch effect by negatively regulating the expression of downstream target genes.

Direction of VSMCs phenotype switching varies depending on Notch receptors. In VSMCs, Notch2 and 3 dominate the expression of Notch receptors, while Notch 1 marginally expressed [99]. Activated Notch1 inhibits cardiomyin-induced VSMCs differentiation via basic helix-loop-helix (bHLH) of HES and HERP that act as repressors of transcription [100]. Earlier studies indicated that activation of RBP-JK by overexpression of Notch1 and Notch3 promoted dedifferentiation of VSMCs [101]. Notch can sense the changes of mechanical stress around VSMCs as a cell membrane receptor and orchestrate cell fate. Given cyclic strain stimulation, VSMCs in vitro down-regulate the expression of Notch3 receptor and target genes, and lose the hyperproliferative phenotype to obtain higher contractility [102]. In vitro, Notch3 expression in VSMCs is also affected by the PDGF-BB and angiotensin 2 [103]. In contrast to Notch3, when JAG-1 binds to Notch1, its NICD binds to miR-143/145 promoter, which greatly up-regulate miR-143/145 to maintain contractive phenotype of VSMCs [104]. Jeremy and colleagues observed that Notch2 decreased and Notch3 increased after PDGF treatment of VSMCs in vitro, and it was further found that Notch2 inhibited VSMCs proliferation while Notch3 up-regulated pro-survival genes through MAPK/ERK pathway [105].

A concern is that Notch pathway is initiated by ligand-receptor interaction among cell–cell contact, but the above findings are mainly based on the gain-of-function or loss-of-function experiment of single VSMCs lineage in vitro. The interaction between VSMCs and other cell types (such as endothelial cells, macrophages, and lymphocytes) in the complex environment of vascular diseases may involve Notch pathway to regulate VSMCs phenotype, which needs to be further investigated.

**Fig. 1:**
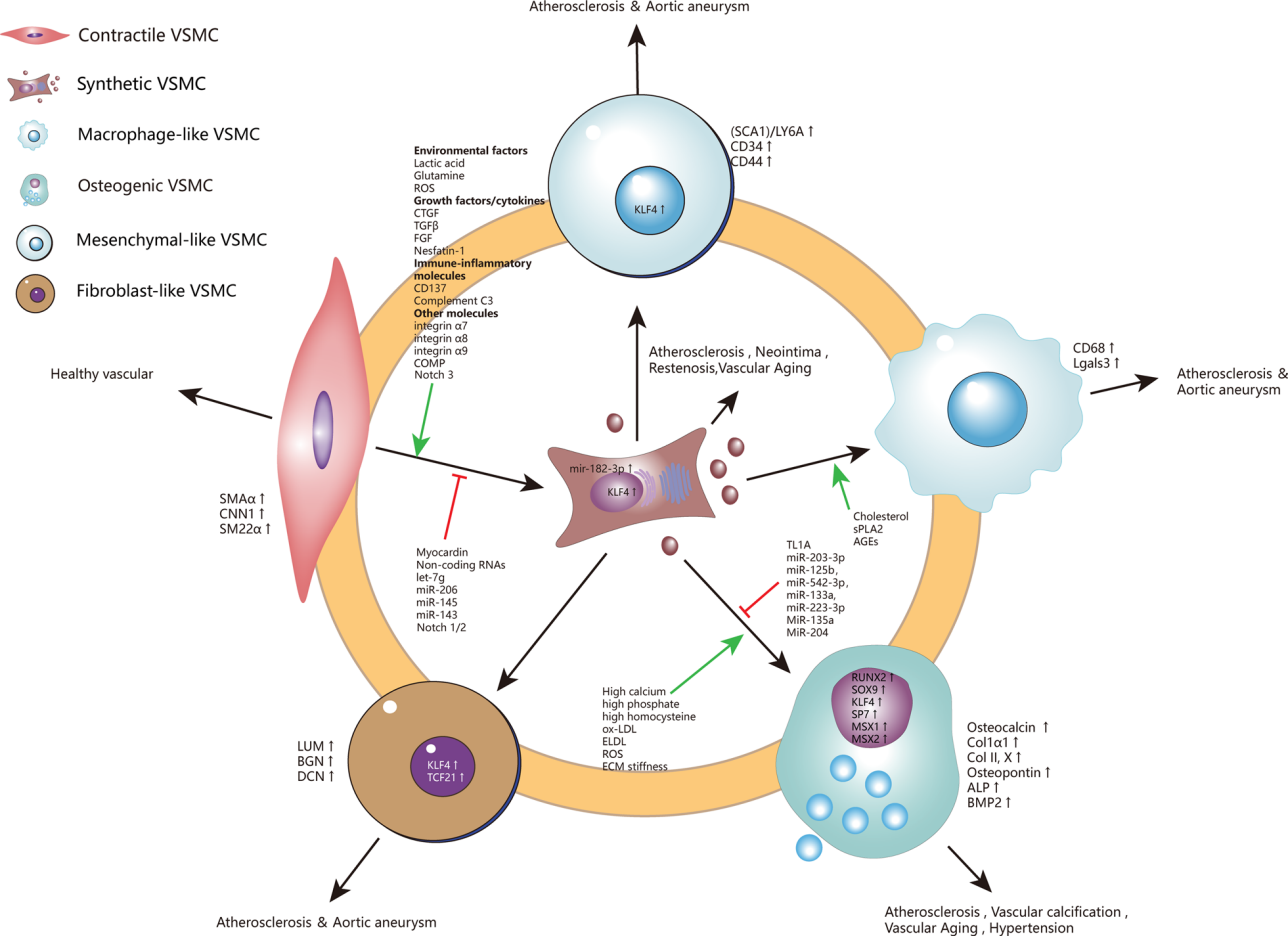
High plasticity of VSMCs. Contractile VSMCs spontaneously modify their phenotype instantaneously to synthetic phenotype when vascular damage occurs. synthetic VSMCs undergo down-regulation of contractile gene expression, cytoskeleton remodeling, and cell reprogramming. Synthetic VSMCs then switch into various phenotypes under a sophisticated regulating network. VSMCs, vascular smooth muscle cells; ECM, extracellular matrix; LGALS3, galectin-3; HSPG, heparan sulfate proteoglycan; ApoB, apolipoprotein B; PDGF, platelet derived growth factor; TNF-α, tumor necrosis factor alpha; IFN-γ, Interferon-gamma; IL-1, Interleukin-1; MMPs, metalloproteases, SMAα, α-smooth muscle actin; CNN1, Calponin 1; SM22α, smooth muscle protein 22α; ROS, reactive oxygen species; CTGF, connective tissue growth factor; TGF-β, transforming growth factor-β; FGF, fibroblast growth factor; COMP, Cartilage oligomeric matrix protein; LUM, lumican; BGN, biglycan; DCN, decorin

At a certain time point, VSMCs populations with seemingly identical phenotypes (same morphology, same marker) may be in different metabolic/transcriptional states, showing a wide variety of potential phenotypes. Thus, when changes in the external environment (physical damage, inflammation, matrix destruction) act on the blood vessel, different populations of VSMCs initiate their own programs to differentiate into distinct phenotypes in response to metabolic changes [106]. Although VSMCs have a variety of phenotypes, the authors believe that VSMCs first dedifferentiated from contractile to synthetic state, and obtained high plasticity before continuing to transform into other pathogenic phenotypes. That's why KLF4 is involved in all phenotype transformation of VSMCs, so KLF4 is like the “master switch” of all VSMCs phenotype. The knowledge of “Factors that contribute to VSMC phenotypes switching” section is summarized in Fig. 1.

## VSMCs phenotype switching and vascular aging

Vascular aging is the basis of vascular dysfunction and vascular diseases. The incidence of AS, AA, hypertension, vascular cognitive impairment, and other vascular diseases all increase with the increase of age. Arterial aging is characterized by thickening and stiffening of the vascular wall and loss of elasticity [107]. Aorta aging may manifest as lumen enlargement, and peripheral artery aging can be clinically characterized by increased ankle brachial index (ABI). Aorta aging can result in loss of its function as a shock absorber to protect the downstream arterioles and visceral arteries from large fluctuations in blood pressure and high blood flow velocity. In the long life-span, gradually accumulated damage factors affect endothelial cell and VSMCs homeostasis, thereby initiating cellular senescence. Senescent cells secrete massive inflammatory mediators and matrix metalloproteinases to induce atherosclerosis [108].VSMCs senescence is characterized by VSMCs phenotype switching and VSMCs stiffness. The following molecular mechanisms are involved in this process: mitochondrial dysfunction, telomere attrition, DNA damage, epigenetic changes, oxidative stress, impaired resistance to molecular stressors, chronic low-grade inflammation, genomic instability, and cellular senescence, and altered intercellular communication in the vascular system [109, 110]. A large part of these molecular mechanisms also contributes to vascular disease by mediating VSMCs phenotype switching.

### VSMCs phenotype changes in vascular aging

As mentioned above, multiple damage factors in the circulation may cause DNA damage to VSMCs. If DNA damage becomes persistent and irreparable, VSMCs may become senescent [111]. In vascular aging process, the endothelial barrier function is disrupted, facilitating circulating harmful substances such as oxLDL to affect VSMCs in tunica media. Another aging mechanism is telomere shortening resulting from excessive VSMCs replication, also named replicative senescence [112]. Senescent cells finally develop into senescence­associated secretory phenotype (SASP), and secrete inflammatory cytokines such as IL-6 [111]. VSMCs are no exception. The role of VSMCs phenotype switching in vascular aging cannot be ignored. A decrease in contractile markers and an increase in synthetic markers OPN were observed in mice models of aging and hypertension [113]. Although this makes SASP-VSMCs look similar to synthetic VSMCs, there are significant differences between the two. Because synthetic VSMCs is the phenotype that present in the early stage after vascular injury, it differentiates into contractile VSMCs after vascular repair has been completed. However, the phenotype transformation of SASP-VSMCs is irreversible. Furthermore, synthetic VSMCs show a decreased expression of skeleton protein and integrin protein, while SASP-VSMCs are accompanied by the increase of cell skeletal protein. It is worth being noted that the expression level of osteogenic gene in the senescent VSMCs is raised (RUNX2, BMP2, ALP), this may be related to aging vascular is prone to calcification [114].

The other changes of senescent VSMCs are VSMCs stiffness and VSMCs adhesion. The non-Muscle actin cytoskeleton of VSMCs enables contractile force to be transmitted to the ECM via integrin-based focal adhesion. Therefore, the number of non-muscle actin cytoskeleton and FA determines the VSMCs stiffness. The expression levels of α-SMA, integrin, and adhesive plaque constituent proteins increased in the VSMCs of elderly monkeys, resulting in more contractile and adhesive forces and increased VSMCs [115]. Zhu et al. found more intensive actin filaments, higher elastic modulus, and higher adhesion force to fibronectin and β1integrin in senescent VSMCs by using the atomic force microscope (AFM) [116]. Aging vascular are prone to form neointima after mechanical injury, this is because angiogenesis-related genes are highly expressed in senescent VSMCs [117]. Milk Fat Globule EGF-8 protein (MFG-E8) is a marker of vascular aging that enhances NF-κB dependent inflammatory response and promotes VSMCs proliferation through Integrin /ERK1/2 signaling [118, 119].

### VSMCs phenotype changes lead to vascular aging

Vascular stiffness is mainly attributed to ECM remodeling. VSMCs phenotype transformation adds a synergistic effect to promote arterial aging. On the one hand, cell senescence promotes the transformation of VSMCs to SASP. SASP-VSMCs secreted a large number of MMPs for decomposing elastin into fragments, synthesized collagen, and promoted its crosslinking. ECM fragments can act as inflammatory chemokines to attract inflammatory cells infiltration and induce the transformation of VSMCs to inflammatory, macrophage-like phenotype. At the same time, ECM degradation also facilitated VSMCs migration and neointimal formation. Age-related ECM changes also affect the mechanical nature. Integrin receptors in cell membrane sense changes in mechanical forces and transmit them to the actin cytoskeleton through the out-inside signaling pathway, which subsequently promotes the dedifferentiation of VSMCs from contractile to synthetic [120].

Certain protective pathways are inhibited in senescent VSMCs. Both WNT3A and WNT5A can protect VSMCs survival, WNT3A upregulated IGF1, WISP 1 and WISP 2 through activating β catenin/TCF to promote VSMCs survival. WNT5A up-regulates WISP 1 by CREB phosphorylation to induce β catenin nuclear translocation [121]. However, WNT5A did not increase phosphorylated CREB level in senescent VSMC. Nicholson et al. developed three decoy peptides that destruct the junctions of cytoskeletal protein (including N-WASP, VASP, talin-Vinculin Interface). All of them significantly reduced VSMCs stiffness in vivo and in vitro, confirming the possibility of targeting cytoskeletal protein junctions for the treatment of vascular sclerosis [122].

### VSMCs phenotype switching and vascular calcification

Another significant structural change of vascular aging is the calcification of tunica intima and tunica media. Since vascular calcification occurs only in arteries but not in veins, it suggests that VSMCs, the specific component of arteries, play an irreplaceable role in arterial calcification. In vitro experiments showed that apoptotic and synthetic VSMCs release vesicles enriched with calcium phosphate in response to elevated extracellular calcium and phosphorus concentrations [123]. High phosphorus or high calcium environment stimulates the VSMCs to an osteogenic phenotype. The characteristics of osteogenic VSMCs and the factors driving their transformation have been described above. Vascular calcification is positively correlated with the severity of vascular aging. The high expression of osteogenic transcription factors such as RUNX2 and SOX9 makes aging vascular tend to form spontaneous calcification.

Inhibiting VSMCs senescence and osteogenic transformation to treat VC has been proved to be effective in animal experiments. As sirtuin 6 (SIRT6) is a conserved NAD + dependent protein deacetylase modulating telomeric chromatin, SIRT6 is closely associated with aging and cardiovascular disease [124, 125]. In vitro, high expression of SIRT6 decreased OPN and OC in VSMCs, while increasing contractile markers. In CKD-induced VC mice model, the results obtained by up-regulating SIRT6 were consistent with in-vitro experiments. SIRT6 reduces the stability of RUNX2 protein through deacetylates RUNX2, and SIRT6 enhances ubiquitination level of RUNX2 for proteasome-dependent degradation [126]. Similarly, Sirtuin 1 (SIRT1) has also been observed to inhibit osteogenic transformation of VSMCs [127]. MiR-34a enhances aortic calcification by directly inhibiting SIRT1 and inducing VSMCs aging [114]. Notably, resident VSMCs in tunica media are not the only source of osteogenic cells. In the CKD model built by Gli1-Creert-mtomato mice, Gli1 + cells migrated to the tunica media and intima and differentiated into osteoblasts (RUNX2 + ALP +), but did not express VSMC marker ACTA2. Gli1CreER^T2^ ± IDTR ± ApoE-/- mice model was constructed to conditionally knock down Gli + cells, which significantly reduced RUNX2 levels and calcified areas [31].

**Fig. 2:**
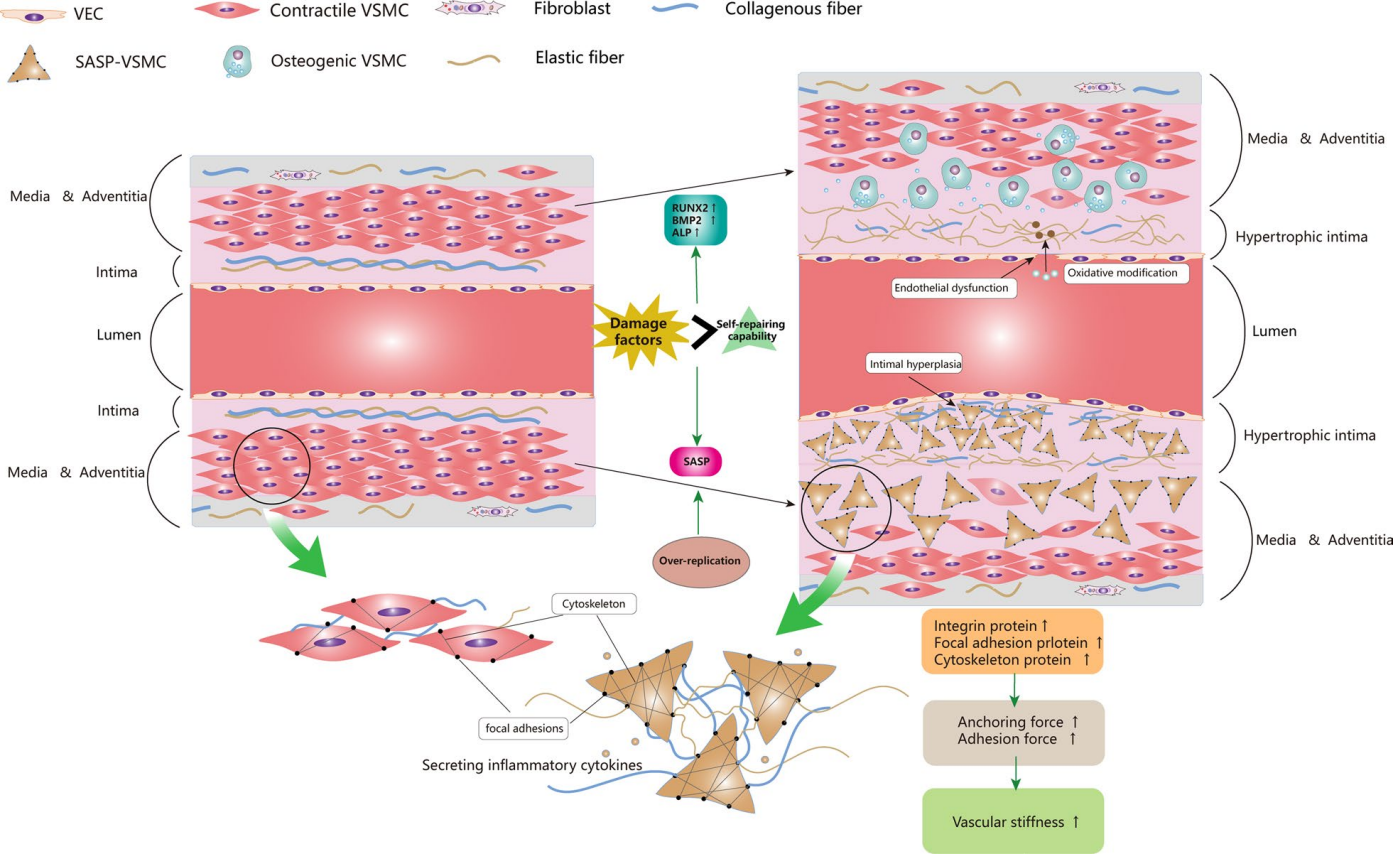
VSMCs phenotype switching and vascular aging. In vascular aging, environmental damage factors trigger mitochondrial dysfunction, telomere attrition, DNA damage, epigenetic changes, oxidative stress, impaired resistance to molecular stressors, chronic low-grade inflammation, genomic instability in VSMCs. Once these damage factors accumulate to a certain extent and cannot be repaired, VSMCs switch to senescence­associated secretory phenotype (SASP). The impaired endothelial barrier caused by vascular aging accelerated the environmental damage on VSMCs. Meanwhile, overproliferation of VSMCs also contributes to SASP. SASP-VSMCs became irregular in morphology and highly expressed integrin protein, focal adhesion protein, and cytoskeleton protein. Cytoskeleton actin was connected to elastin and collagen in ECM through integrin-based focal adhesion, delivering anchoring force and adhesion force. When the above protein is up-regulated, not only increases the VSMCs stiffness but also increases the interaction between ECM and VSMCs, leading to increased vascular stiffness. SASP-VSMCs secrete a large amount of MMPs for decomposing elastin into fragments, synthesize collagen, and promote its crosslinking. This process increases ECM stiffness and decreases ECM elasticity. On the other hand, the high expression of RUNX2, BMP2, and ALP in senescent VSMCs makes it susceptible to switch to osteogenic VSMCs under high calcium or phosphorus environment. Osteogenic VSMCs release calcium phosphate enriched vesicles to promote VC. VEC, vascular endothelial cell; SASP, senescence­associated secretory phenotype; RUNX2, runt-related transcription factor 2; BMP2, bone morphogenetic protein-2; ALP, alkaline phosphatase; VC, vascular calcification

Vascular aging and calcification are generally considered irreversible processes, and the in-depth understanding of phenotype switching of VSMCs provides us with potential prevention and treatment strategies. Elimination of metabolic risk factors is always a priority, this includes correcting calcium and phosphorus metabolism disorders and lowering LDL homocysteine ROS levels. Secondly, transcription factors that drive VSMCs ossification and molecules that promote VSMCs senescence can be directly inhibited, or the osteogenic genes can be indirectly regulated by miRNAs. Animal studies have demonstrated the feasibility of these approaches, but more evidence is needed before clinical use. The key to treatment is how to specifically and precisely regulate the phenotype transformation process. In addition, VSMCs phenotype switching is intended to repair vascular damage, so the long-term outcome of inhibiting the process remains to be further studied. The knowledge of “VSMCs phenotype switching and vascular aging” section is summarized in Fig. 2.

## VSMCs phenotype switching and atherosclerosis

Acute or chronic ischemia of vital organs due to atherosclerosis (AS) remains the leading cause of death and disability worldwide [128, 129]. The nature of AS is the chronic inflammation caused by sophisticated interactions between multiple cell types (including macrophages, VSMCs, endothelial cells, monocytes, and lymphocytes) in vascular wall [130, 131]. VSMCs in normal tunica media express a series of contractile markers, such as α-actinin-2 (ACTA2/α-SMA), transgelin (TAGLN/SM22α), smooth muscle cell myosin heavy chain 11 (MYH11/SMMHC), and smooth muscle cell differentiation specific antigen (Smoothelin/SMTN) [132]. However, in AS lesions, VSMCs express markers commonly expressed in macrophages, mesenchymal stem cells, foam cells, and osteochondrocytes [11, 15, 19, 27, 133, 134]. Furthermore, the migration and proliferation capacity of VSMCs were enhanced, and more ECM proteins and cytokines were secreted [135].

### Lineage-tracing studies revealed VSMCs phenotype switching in AS

Early in the 1970s, Ross and Campbell et al. discussed the VSMCs phenotype change in AS lesions and guessed that the pathogenic VSMCs migrated from the contractile VSMCs in tunica media [136, 137]. In vitro, 15% of cultured cells from normal arteries expressed a chimeric phenotype, while 25% of cells from adipose streaks expressed a chimeric phenotype [18]. This suggests that in AS lesions, intracellular lipid accumulation and other stimulators can activate the expression of macrophage-associated antigen CD68 in VSMCs. The approach to identifying cell types by specific markers has drawbacks, as the expression of some cell markers may be lost during disease progression. Shankman et al. labeled mouse VSMCs with YFP and found that approximately 82% of VSMCs-derived cells (YFP+) in AS lesions did not express the contractile marker ACTA2 [27]. Among all VSMCs derived cells, macrophage-like cells accounted for 30%, MSC-like cells accounted for 7%, myofibroblast-like cells account for 12%, and uncertain cell phenotypes account for 32–51% [27]. This study suggests that the majority of VSMCs in AS lesions cannot be identified by conventional SMC markers and that cells previously thought to be components of macrophage fibroblasts and mesenchymal stem cells may be incorrect.

Using a combination of in situ hybridization (ISH) and proximity ligation assays (PLA), Gomez et al. found that dimethylation of lysine 4 of histone H3 (H3K4me2) at the MYH11 locus was specific in human and mouse VSMCs, it even exists in VSMCs lacking contractile markers in AS lesions [138]. The study indicated that these chimeric cells were derived from VSMCs. However, the conclusion was challenged by the work of Mariana et al., as they found that H3K4me2 at the MYH11 locus also existed in SCA1 + vascular stem cells [139]. Feil et al. used Sm22-ER^T2^Cre-lacZ ApoE^−/−^ mice to trace VSMCs lineage and found that VSMCs-derived cells activated macrophage markers (LGALS3 and CD68) in advanced AS plaque [19]. In 2016, Chappell et al. prelabeled mature VSMCs in ApoE^−/−^ mice arteries using multicolor lineage labeling and specific conditional lineage tracing techniques, and subsequently observed partial VSMCs switched into MAC3 + macrophage-like cells that was not macrophage-derived [133]. Although the lineage-tracing techniques and in situ hybridization techniques could accurately identify the origin of these chimeric cells, the amount of evaluable VSMCs phenotype is limited.

Using single-cell transcriptome sequencing and lineage-tracing technique, Wirka et al. found that LGALS3 + VSMCs were first transformed into fibroblast-like cells in AS lesions, which is termed "fibromyocytes" [35]. Fibromyocytes were also termed by Wirka et al. as ECM remodeling pioneer cell phenotype. However, the study has its limitations since the cells used for analysis were mostly from tunica media and tunica adventitia rather than the atherosclerotic area. In addition, Wirka et al. believed that fibromyocytes could produce protective fibers to form FC, which was beneficial in maintaining plaque stability. However, in the experimental studies, the deletion of KLF4 and OCT4 resulted in the thickening of the fibrous cap, which may suggest that the expression level of KLF4 and OCT4 is negatively correlated with the number of fibromyocytes [27, 140]. LGALS3 + VSMC-derived cells present a stem-cell-like, ECM-remodeling phenotype, and LGALS3 + VSMCs subsequently transform into three other VSMCs phenotypes (e.g., osteogenic phenotypes) in advanced lesions, which may contribute to plaque calcification and plaque instability [23]. Pan et al. suggested that VSMCs in AS transitioned into an intermediate cellular state and called them SEM cells (SEM = stem cell + endothelial cell + monocyte). SEM cells were pluripotent and could differentiate into macrophage-like and fibrochondrocytes, or return to a contractile phenotype [24].

### VSMCs phenotype switching and foam cell formation in AS

Lipid deposition and foam cell formation are the characteristic pathological feature of AS. The pathological results showed that numerous foam cells exist in the lipid-rich necrotic core. It was previously believed that these foam cells were derived from bone marrow-derived macrophage that have engulfed lipid and necrotic cell debris. A recent lineage tracing experiment exploring the origin of foam cells in AS showed that manifold VSMCs, which should be quiescent in the tunica media, migrated to tunica intima and transform to macrophage-like VSMCs [132]. A recent study showed that in advanced coronary atherosclerotic plaque, 50% of foam cells express the VSMCs marker ACTA2. On the other hand, most ACTA2 + foam cells also expressed the macrophage marker CD68, accounting for 40 percent of all CD68 + cells [141].

In respect of the function of macrophage-like VSMCs, they have a similar capacity to macrophages and can also engulf lipid and necrotic cell debris to form foam cells. However, the phagocytosis of macrophage-like VSMCs is worse than that of myeloid-lineage macrophages, and their presence is thought to reduce the efficiency of lipid disposal and debris clearance in plaques, thereby exacerbating the formation of atherosclerotic plaques [132]. Another study showed that VSMC-derived foam cells carried a higher cholesterol burden than leukocyte derived foam cells [142]. The lower ABCA1 expression level render VSMC-derived foam cells incapable of cholesterol efflux, so that VSMC is more likely to form foam cells than macrophages [141, 142].

### VSMCs phenotype switching and unstable plaque rupture in AS

Unstable plaque rupture and subsequent thrombotic events are the primary causes of acute ischemia (e.g., ischemic stroke and myocardial infarction) caused by AS [143–145]. The results of autopsy and radiographic studies indicate that stable plaques have thicker fibrous caps enriched with collagen and a smaller necrotic core compared to unstable plaques [145, 146]. The fibrous cap consists of collagen-proteoglycan matrix and VSMCs, with the infiltration of macrophages and lymphocytes. Thinning of fibrous cap and local inflammation lead to increased plaque instability [147]. Reduced collagen synthesis and enhanced ECM degradation in plaque cells (mainly smooth muscle cells) are considered to be the principal reasons for the thinning fibrous cap. ACTA2+ cells were the main cellular components in the fibrous caps of stable plaques, while there were more CD68/LGALS3 positive cells in the fibrous caps of unstable plaques. Some of these macrophage-like cells were from myeloid lineage, and some were from VSMCs [27].

How to maintain AS plaque stability by regulating VSMCs phenotype switching has become a hot issue. KLF4 is a candidate therapeutic target as previously described. Following specific knockout of KLF4 of VSMCs, the size of AS lesions is reduced by nearly 50% while the size of fibrous cap is increased by more than twice; the percentage of ACTA2+ cells is increased while the percentage of LGALS3+ cells in the fibrous cap has been significantly decreased [27]. In addition, SMC-specific KLF4 knockout did not change the number of VSMC-derived cells but changed the proportion of ACTA2 + cells and the proportion of other VSMC-derived cells (including SCA1+ cells and LGALS3+ cells). These results suggest that KLF4 deletion may play a beneficial role in plaque development by inhibiting the transformation of VSMCs into macrophage-like VSMCs and mesenchymal-like VSMCs. Octamer-binding transcriptional factor 4 (OCT4) maintains the pluripotency of embryonic stem cells (ESCs) [148]. Cherepanova et al. [140] found that OCT4 was activated in mouse and human atherosclerotic lesions, and VSMC-specific OCT4 knockout led to larger AS lesions and decreased plaque stability (including thinning of fibrous caps, increased necrotic core, and increased intraplaque hemorrhage). VSMCs-specific OCT4 knockout also result in less ACTA2+ and more LGALS3+ VSMCs in AS lesion, which was completely in contrast to the result after KLF4-knockout [140]. TCF21 is a causal coronary artery disease gene. In cultured human coronary artery SMCs (HCASMCs), the downregulation of TCF21 led to the up-regulation of VSMCs contractile markers. Wirka et al. found that the TCF21 deficiency resulted in the reduced fibromyocytes in the fibrous cap and inhibited VSMCs phenotype switching, hence it was speculated that TCF21 might play a protective role by promoting the migration of fibromyocytes into AS lesion and fibrous cap [35].

However, the pathological proliferation of smooth muscle cells (SMCs) like cells in the intima and media were not all from the resident medial VSMCs. Adventitia contains abundant stem/progenitor cells (marked by Sca1, CD34, C-kit, and Flk1) such as AdvSca1 cells that could reprogram into SMCs, endothelial cells (ECs), myofibroblasts, macrophage-like cells, chondrocytes, and adipocytes, involved in the pathogenesis of atherosclerosis [25]. Injured VSMCs highly express and secrete aortic carboxypeptidase-like protein (ACLP) [149], that promote the differentiation of AdvSca1 cells into myofibroblasts (ACTA2+ COL1A1+) partially through MRTFA pathway [150]. The differentiation of progenitor cells into SMC-like cells is also regulated by PDGF-BB receptor, TGFβ receptor and Integrins. Therefore, it is necessary to elucidate the extent of contribution of VSMCs in tunica media and AdvSca1 cells to AS. In ApoE^−/−^ mice, SCA1+ cells accounted for 2.3% of the total cell number in AS lesion which may be attributed to the loss of markers during the differentiation of progenitor cells [151]. Through the SCA1-CreERT/Pdgfra-DreERT-mTomato lineage tracing mice, labeled SCA1+ PDGFRa+ cells accounted for 13% of the total SMCs of neointima in the mouse model of femoral artery injury. The Gli1-CreER^T^-mTomato lineage tracing system used by Kramann et al. in the AS-CKD model showed that, after 16 weeks, approximately 20% of the cells in the media and nearly 50% of the cells in the intima were derived from GLI1+ progenitor cells. But the cells differentiated by GLI1+ macrophages did not express CD68, indicating that macrophage-like VSMCs is independent of the outer membrane progenitor cells [31]. Conditional knockout of GLI+ cells could not reduce plaque size and the number of macrophage-like cells. Mayr et al. used PDGF-BB to induce AdvSca1 cells to differentiate into SMC-like cells, and subsequently found that the protein expression profile of these SMC-like cells was similar to VSMCs of ApoE^−/−^ mice but not WT mice. These cells were highly sensitive to oxidative stress, indicating their high susceptibility to AS [151]. Functionally, differentiation from AdvSca1 cells into SMC-like cells increases the overall contractile force of the vessel, whereas the switching of VSMCs from contractile to multiple phenotypes is a process of loss of contractile force. Thus, compensation of contractile force may have occurred in the process.

### VSMCs phenotype switching and neointima in AS

The early stages of AS involve neointima formation primarily consisting of VSMCs and ECM (such AS proteoglycan and elastin, etc.) [147, 152]. VSMCs can promote intima thickness through their proliferation and secretion of ECM (including elastin collagen and proteoglycan, etc.). Chappell et al. showed that VSMCs-derived cells in the neointima of AS were usually formed by clonal expansion of a few VSMCs in tunica media [133]. CD137 belongs to the tumor necrosis factor receptor superfamily (TNFRSF), including CD40 LIGHT and OX40, and plays a vital role in atherosclerosis [153, 154]. CD137 can induce neointima as the VSMCs of neointima are highly expressed NFATc1 and vimentin, low expressed MYH11 and α-SMA, implying its transformation to synthetic VSMC. In ApoE^−/−^ mice, NFATc1 deletion reduced CD137L-induced neointima formation [155].

**Fig. 3:**
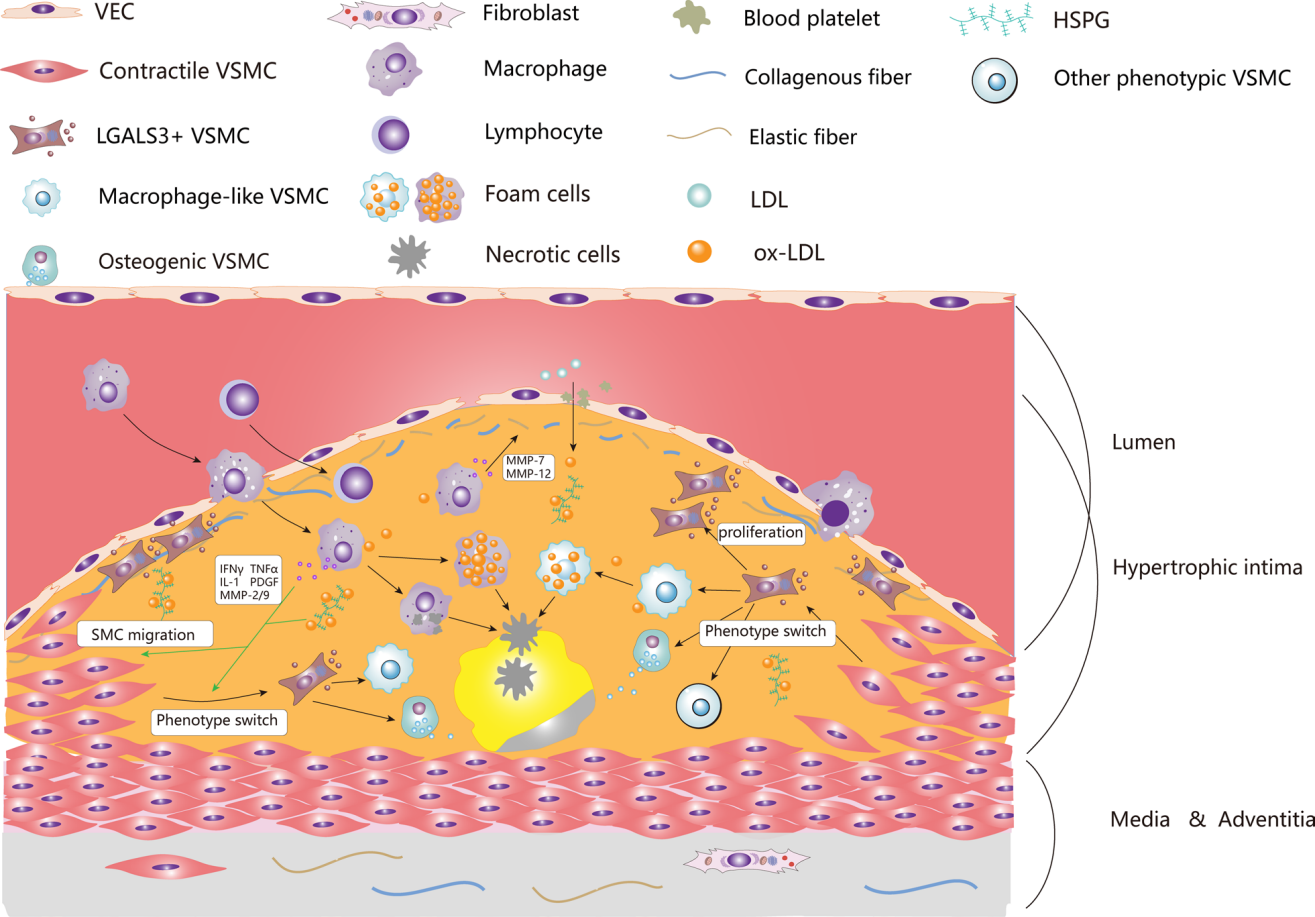
VSMCs phenotype switching in advanced atherosclerotic plaque. Due to damage or dysfunction of endothelial cells, circulating LDL (including ApoB) passes through the endothelial barrier into the subendothelium and is subsequently captured by HSPGs in the ECM. Subsequently, LDL is gradually oxidized into ox-LDL under free radicals or other oxidants. The accumulation of ox-LDL induces the migration of macrophages and monocytes to the lesion area. Macrophages engulf oxidized lipids and necrotic cells in the lesion, secrete inflammatory cytokines, and transform to foam cells. Finally, these foam cells are apoptotic and coalesce into a lipid-rich necrotic core in subendothelium. Macrophage-like VSMCs also engulf lipid and necrotic cell debris to form foam cells. However, the phagocytosis of macrophage-like VSMCs is lower than that of macrophages, disrupting the efficiency of lipid disposal and debris clearance in plaques, thereby exacerbating the formation of atherosclerotic plaques. Meanwhile, ox-LDL and various inflammatory cytokines (such as PDGF, TNF-α, IFN-γ, IL-1, MMP-2/9, etc.) can stimulate VSMCs to migrate from tunica media to tunica intima to form neointima and phenotype transformation occurred in the process The contractile VSMCs first transform into LGALS3 + VSMCs, this kind of cell is also known as pioneer cells, Stem/Endothelial/Macrophage (SEM) cells, or fibromyocytes. These transitional VSMCs can synthesize ECM, participate in the formation of fibrous cap, and can also transform into macrophage-like VSMCs, osteogenic VSMCs, and other types of VSMCs. Macrophage-like VSMCs derived foam cells secrete MMPs to destroy ECM in the fibrous cap, and osteogenic VSMCs participate in calcification of lipid necrotic core, both of which are generally thought to aggravate plaque instability. VEC, vascular endothelial cell; VSMC, vascular smooth muscle cell; ECM, extracellular matrix; LGALS3, galectin-3; LDL, low density lipoprotein; ox-LDL, oxidized low-density lipoprotein; HSPG, heparan sulfate proteoglycan; ApoB, apolipoprotein B; PDGF, platelet derived growth factor; TNF-α, tumor necrosis factor alpha; IFN-γ, interferon-gamma; IL-1, interleukin 1; MMPs, metalloproteases

Because of the difficulty of directly tracking VSMCs in human AS lesions, the lineage tracing experiments were carried out in vivo or in-vitro cultured cells, which confirmed the VSMCs phenotype switching in AS to some extent. In addition, VSMCs are thought to achieve phenotype switching through selective expression of marker genes. But some cellular proteins and mRNAs can be transferred between cells via exosomes or microvesicles so that VSMCs can obtain markers in a passive way. Furthermore, the contribution of endogenous progenitor cells in the tunica media or adventitia remains to be further verified. The knowledge of “VSMCs phenotype switching and atherosclerosis” section is summarized in Fig. 3.

## VSMCs phenotype switching and aortic aneurysm

The most typical and severe dilated artery disease is aortic aneurysm (AA) including thoracic aortic aneurysm (TAA) and abdominal aortic aneurysm (AAA), reputed for its high risk of death. AA is commonly defined as a permanent and localized dilatation of the aorta exceeding 150% of the original diameter [156]. Common risk factors for AA formation include males, smoking, the elderly, dyslipidemia, hypertension, and obesity [157–159]. The direct cause of AA formation is the destruction of structural integrity of aorta and decreased arterial compliances, and the pathophysiological basis is ECM degradation, leukocytic infiltration, and apoptosis/dysfunction of VSMCs [157, 160, 161]. The damage of arterial structural integrity means a weak aortic wall. As the supporting force in aorta is mainly provided by the intermediate smooth muscle layer, AA formation can be associated with VSMCs apoptosis and impaired VSMCs proliferation. ECM degradation also contributes to a weak aortic wall. Studies have found that elastase [162], collagenase [163], and MMPs secretion are increased in AA [164]. The main components of ECM, fibronectin, laminin, collagen, and elastin was connected to VSMCs by integrin-based focal adhesions and transferred anchorage and traction forces, providing structural support [165–167]. In AA, the protease caused extensive ECM degradation, and the resulting ECM remodeling damaged the mechanical connection between ECM and VSMCs. As a result, the aorta loses its overall structural integrity and intrinsic molecular junctions. Additionally, the tunica media of aorta consists of serval concentric elastic lamellae layers and VSMCs layers, and they act in concert to provide the compliance and recoil properties of the aortic wall [168]. Concentric elastic lamellae is not visible in the human AA and animal AA models but fragmentation of elastic fibers.

Until now, studies investigating pharmacological treatment for AA have generally failed, due to insufficient understanding of the causes of AA. Early studies suggested that atherosclerosis and aortic mural thrombosis may be the causes of AA formation [169, 170]. The growth of atherosclerotic plaque causes compensatory enlargement of the total aortic cross-sectional area, thus maintaining sufficient lumen size. Most drugs currently used for secondary prevention of atherosclerotic disease have been shown to reduce cardiovascular events in patients with AAA [157]. In FAME-2 Trial (ACTRN12613001039774), however, Fenofibrate did not significantly reduce AAA growth rate or AAA serum marker concentration [171]. A meta-analysis reports that the idea that statin therapy reduces AAA expansion rates is based on low-quality clinical evidence, and was not significant in meta-analyses based on high-quality studies [172]. Human AAA derived intraluminal thrombus contains a range of inflammatory cells (particularly neutrophils) and their products (such as matrix metalloproteinases and proinflammatory cytokines), which is associated with AAA progress. So far, there has been no report on the impact of novel oral anticoagulants on AAA growth. A multicenter randomized controlled trial (NCT02070653) involving 144 participants demonstrated that Ticagrelor did not reduce the expansion rate of small AAA [173]. At the same time, synthetic inhibitors of MMPs (Batimastat, Marimastat) and tetracycline derivatives (doxycycline hydrochloride) have shown good results in animal studies, but clinical trials did not favor its efficacy and safety [174].

Therefore, the researchers turned their attention to new mechanisms for AA formation. VSMCs phenotype switching has provided a new perspective for understanding the occurrence and progress of AA. Single-cell sequencing analysis revealed the presence of contractile VSMCs, mesenchymal-like VSMCs, macrophage-like VSMCs, and fibroblast-like VSMCs in TAA and AAA [34, 175]. The latest research has identified VSMCs phenotype switching as an underlying destructive factor. Part of VSMCs in TAA and AAA is transformed from contractile to synthetic, which has been confirmed by manifold clinical and animal studies: in human AA tissues and animal AA models, the mRNA and protein level of contractile markers were decreased, while the level of OPN, MMP-9, ELK-1, and Vimentin were increased [176]. Theoretically, the pathophysiological mechanism of synthetic VSMCs in AAA has been thoroughly studied: (1) The reduced contractile force of synthetic VSMCs prevents the aorta from contracting to its original shape after expansion in pulse pumping blood, resulting in permanent diameter increase; (2) The synthetic VSMCs exert a significantly increased MMPs secretion capacity, which disrupted concentric elastic lamellae and the mechanical connection between VSMCs and ECM, resulting in reduced aortic compliance; (3) Synthetic VSMCs secrete inflammatory factors and chemokines that mediate immune cell infiltration into the aortic wall, further aggravating chronic inflammation; (4) The number of integrin-based focal adhesion on the surface of synthetic VSMCs decreased, resulting in inadequate anchorage and traction forces between VSMCs and ECM.

Both contractile and synthetic genes are up-regulated in MFS-induced AA, which is different from normal AA, because single-cell RNA sequencing showed an increased proportion of synthetic VSMCs and an increase in the overall number of VSMCs [177]. TGF-β signaling pathway dysregulation contributes to the formation of AA in MFS patients [178]. There are many downstream signaling pathways of TGF-β, and they are antagonistic to each other in the regulation of VSMCs phenotype, the classical SMAD pathway also promoted the expression of the contractile and synthetic marker, while the non-classical ERK pathway inhibited the expression of these markers [177]. Conversely, another in-vitro study showed that ERK1/2 promote the transformation of VSMCs to synthetic phenotype. The differences may be due to the unique pathogenesis of MFS [54].

**Fig. 4:**
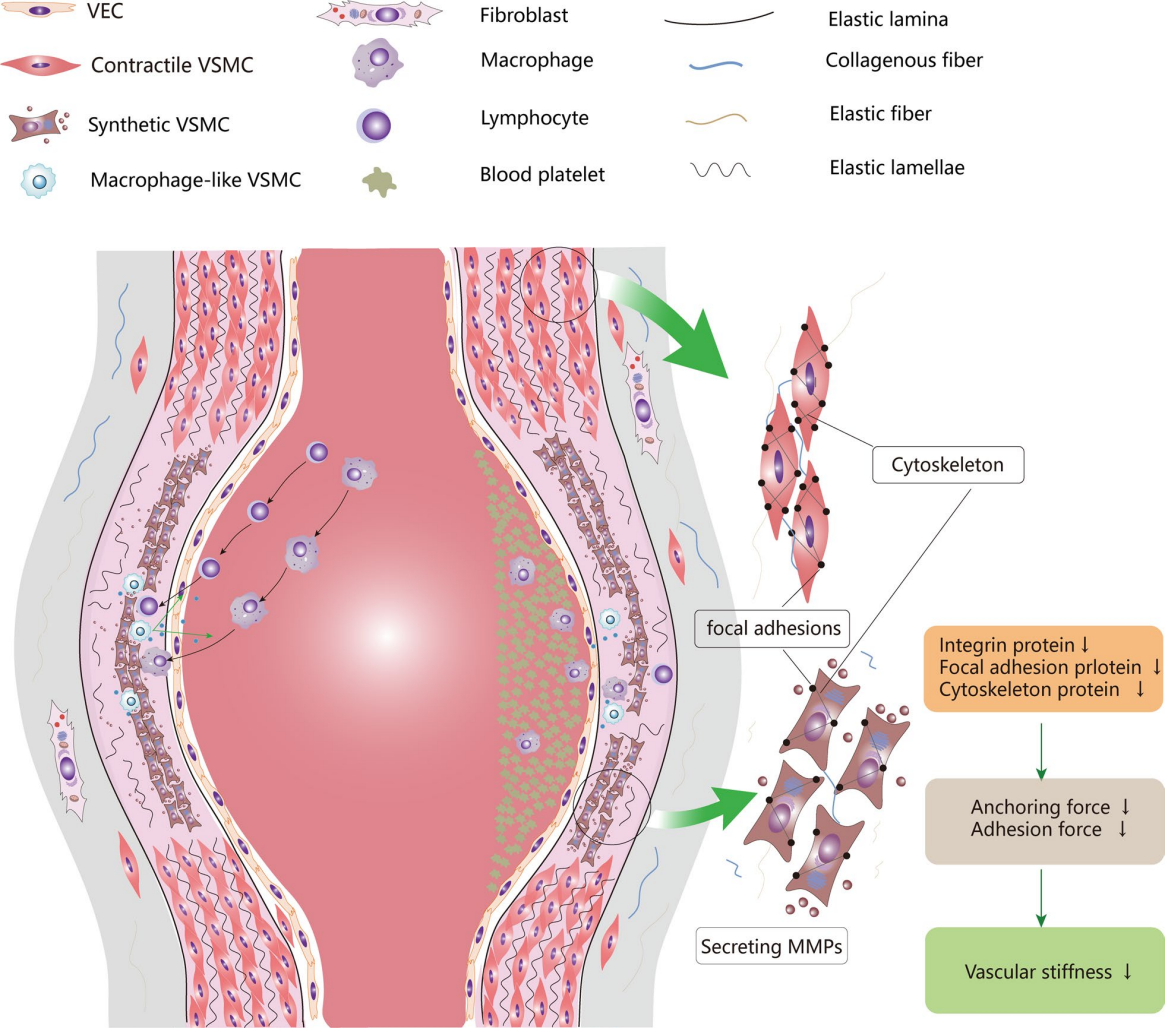
VSMCs phenotype and aortic aneurysm. The aortic wall comprises tunica intima, tunica media, and tunica adventitia. Tunica intima consists of a single layer of endothelial cells and basement membrane. Tunica intima and tunica media are separated by internal elastic lamina. Tunica media is composed of multilayer annular elastic lamellae and annular arranged VSMCs and is filled with collagen-rich and elastin-rich ECM. The tunica adventitia is composed of collagen, elastic fiber, and fibroblast, separated from the tunica media by the external elastic lamina. VSMCs and elastic fibers in tunica media provide active and passive contractility respectively. In aortic aneurysm, VSMCs switch to synthetic phenotype, down-regulate contractile protein, and secret massive MMPs and inflammatory cytokines. Down-regulated contractile proteins mean that the aorta cannot provide enough contractile force to restore its original diameter during pulse pumping. Moreover, the abundant collagen and Elastin in the aortic ECM were decomposed into fragments by MMPs, resulting in disrupted elastic lamina and elastic lamellae. Therefore, the aorta does not generate sufficient passive contractile force in dilated condition. As synthetic VSMCs express less integrin protein, focal adhesion protein, cytoskeleton protein, the anchoring force between VSMCs and ECM decreases, resulting in weaker passive contraction force. Macrophage-like VSMCs, on the other hand, secrete inflammatory factors and chemokines, recruit monocytes, macrophages, and lymphocytes to infiltrate into the aortic wall. These cells form chronic inflammation and participate in the destruction of aortic wall. VEC, vascular endothelial cell; VEC, vascular endothelial cell; MMPs, metalloproteases; ECM, extracellular matrix

It is believed that the loss of the contraction ability of VSMCs because of VSMCs phenotype switching is the mechanism of AA. However, as synthetic VSMCs are usually a reaction after injury, the possibility that VSMCs phenotype transformation is a compensatory mechanism after AA formation cannot be ignored. VSMCs have a strong capability of proliferation in the synthetic state, which counters the pathological features of VSMCs apoptosis. Therefore, synthetic VSMCs may be a double-edged sword. Although it aggravates AA by secreting MMPs and inflammatory factors, it proliferates to thicken the smooth muscle layer to prevent AA rupture. Single-cell transcriptome sequencing showed macrophage-like VSMCs, mesenchymal-like VSMCs, and fibroblast-like VSMCs in AA. Mesenchymal VSMCs and fibroblast-like VSMCs may play a repairing role in AA, but their specific function needs further investigation. Macrophage-like VSMCs acts an important role in vascular tissue homeostasis and efferocytosis [17]. In AS, macrophage-like VSMCs are induced by oxLDL and transformed into foam-like cells, but this mechanism does not exist in AA. Therefore, we hypothesized that macrophage-like VSMCs accelerates AA progression by mediating immune reaction. Pathological studies of AA showed that infiltration of innate immune cells (such as macrophages, neutrophils, and dendritic cells) and adaptive immune cells (such as B cells and T cells). Macrophage-like VSMCs not only present phagocytosis, but also actively participates in the interaction with immune cells and initiate them migrate into aortic wall. Macrophage-like VSMCs express inflammatory adhesion molecules including TNF-α, IL-1b, IL-6, IL-8, IL-17, CCL2, CCL7, and are involved in chronic inflammatory processes [17, 21, 47]. CCL2/CCR2 can further recruit monocytes and lymphocytes to infiltrate the aorta and aggravate chronic inflammation. The knowledge of “VSMCs phenotype switching and aortic aneurysm” section is summarized in Fig. 4.

## Limitations in studies of VSMCs phenotype switching

Current studies on multiple VSMCs phenotypes rely on the accurate mapping ability of Cre-loxP lineage tracing system. Markers of VSMCs lineage including MYH11, CNN1, SM22α, and ACTA2 were used to drive Cre recombinant enzyme expression. However, precisely target VSMCs is challenging because some VSMCs markers are also expressed in other cell types, such as fibroblasts, myofibroblasts, and endothelial-to-mesenchymal transition. MYH11 is now widely recognized as the most specific marker of smooth muscle cell lineage. Cre should be specifically expressed in VSMCs, and even minute Cre expression in non-VSMCs will disturb the accuracy of lineage tracing. Intraperitoneal injection of tamoxifen dissolved in peanut oil has been reported to increase the spontaneous fluorescence of peritoneal macrophages compared with oral tamoxifen, resulting in false positive results [179]. While using Myh11-CreER^T2^ mice to trace the origin of proliferating SMCs in pulmonary arteriole and alveoli under hypoxic conditions, Sheikh and colleagues mislabeled some SMC-free markers lung cells [180]. Therefore, more precise techniques are needed to elucidate the fate of VSMC-derived cells in vascular diseases. A novel lineage tracing technology Dual-recombinase-activated lineage tracing (DeaLT) that add a Dre-Rox system to control Cre-loxP system that effectively avoid the nonspecific expression of Cre [181].

VSMCs phenotype switching is a critical response to vascular injury stimulus. During this process, VSCs located in adventitia and media also undergo reprogramming and migrate to the intima, cooperating with the VSMCs-derived cells to promote disease. Therefore, the primary goal is to determine their respective contribution to vascular lesions. Meanwhile, intercellular communication between these two cellular populations is highly likely to occur, promoting or inhibiting each other's reprogramming and migration. In addition, it should be realized that phenotype switching is not the only response to injurious stimuli, and apoptosis or death is also the potential fate of VSMCs. For instance, ROS is not only a stimulator of VSMCs phenotype switching, but also an inducer of autophagy of VSMCs. The study by Wang et al. preliminarily demonstrated the relationship between autophagy and phenotype switching in VSMCs [182]. However, the detailed relationship between phenotype switching and other responses of VSMCs remains unclear. After all, it is a complex event that is coordinated with cell fate determination.

## Summary and expectation

Great progress has been made in the study of the role of VSMCs phenotype transformation in vascular diseases in the past 20 years. VSMCs phenotype switching provides a new perspective for understanding the pathogenesis of vascular diseases. The high plasticity of VSMCs is the basis of self-repair after vascular injury. Under normal physiological conditions, this phenotype switching for repair is reversible. However, long-term changes in metabolic factors deviate the phenotype switching direction to pathogenesis and eventually becomes an irreversible process. Disturbances in extracelluar environment caused by chronic diseases also stimulate phenotype changes in other vascular components, such as endothelial cells and ECM. They synergistically aggravate vascular disease with VSMCs phenotype switching.

Importantly, the studies of VSMCs phenotypes provide new ideas and targets for pharmacological treatment. Pharmacological treatments for AS are still old-fashioned lipid-lowering, anti-platelet, and anti-hypertensive regimens. Pharmacological treatments for AA have failed since they were exploited against traditional targets. Currently, VC can only be slowed by controlling risk factors. For these life-threatening vascular diseases, existing treatments cannot reverse their aggravation. Due to the strong plasticity of VSMC, it is feasible to treat vascular diseases by reversing VSMCs phenotype to contractile. As the master switch that controls VSMCs switch from contractile to all pathogenic phenotypes, KLF4 may be the best therapeutic target. Since a large population of miRNAs has similar roles in regulating the VSMCs phenotype, vesicles with several miRNAs can be attempted to reverse the VSMCs phenotype at the lesion site.

Another future challenge is to accurately manipulate VSMCs phenotype switching to not only prevent vascular disease, but also preserve its function in repairing vascular damage. This depends on a rigorous understanding of the biology of each VSMCs phenotypes and their relationship with vascular diseases. The same phenotypic VSMCs plays distinct roles in different stages of disease. In the early stage of a healthy artery, the synthetic VSMCs can lead to a decrease in aortic contractility and a tendency to dilate the aorta; in the advanced stage of AA, the proliferation of synthetic VSMCs can increase the thickness of the middle membrane and prevent AA rupture. It may be a better strategy to firstly induce synthetic VSMCs to proliferate to an appropriate number and then convert it into contractile VSMCs, so that the aorta can obtain both strong contractile ability and thick tunica media. In summary, we still lack a comprehensive understanding of VSMCs phenotype switching and the ability to precisely manipulate it.

## Data Availability

No new data were generated or analyzed in support of this review.
